# Eosinophilic esophagitis—established facts and new horizons

**DOI:** 10.1007/s00281-021-00855-y

**Published:** 2021-06-07

**Authors:** Luc Biedermann, Alex Straumann, Thomas Greuter, Philipp Schreiner

**Affiliations:** 1 Department of Gastroenterology and Hepatology, University Hospital Zurich, Zurich, Switzerland; 2 Division of Gastroenterology and Hepatology, Centre Hospitalier Universitaire Vaudois (CHUV) and University of Lausanne, Lausanne, Switzerland

**Keywords:** Eosinophilic esophagitis (EoE), Epidemiology, Environmental risk factors, Esophageal eosinophilia (EE), Clinical manifestation, Food allergy, Variant-EoE (?)

## Abstract

Despite dramatic advances in our understanding of the pathogenesis and course of disease in the relatively short timeframe since the discovery and first description of eosinophilic esophagitis (EoE) less than three decades ago, many open questions remain to be elucidated. For instance, we will need to better characterize atypical clinical presentations of EoE and other forms of esophageal inflammatory conditions with often similar clinical presentations, nut fulfilling current diagnostic criteria for EoE and to determine their significance and interrelationship with genuine EoE. In addition, the interrelationship of EoE with other immune-mediated diseases remains to be clarified. Hopefully, a closer look at the role of environmental factors and their interaction with genetic susceptibility often in context of atopic predisposition may enable identifying the candidate substances/agents/allergens and potentially earlier (childhood) events to trigger the condition. It appears plausible to assume that in the end—comparable to current concepts in other immune-mediated chronic diseases, such as for instance inflammatory bowel disease or asthma bronchiale—we will not be rewarded with the identification of a “one-and-only” underlying pathogenetic trigger factor, with causal responsibility for the disease in each and every EoE patient. Rather, the relative contribution and importance of intrinsic susceptibility, i.e., patient-driven factors (genetics, aberrant immune response) and external trigger factors, such as food (or aero-) allergens as well as early childhood events (e.g., infection and exposure to antibiotics and other drugs) may substantially differ among given individuals with EoE. Accordingly, selection and treatment duration of medical therapy, success rates and extent of required restriction in dietary treatment, and the need for mechanical treatment to address strictures and stenosis require an individualized approach, tailored to each patient. With the advances of emerging treatment options, the importance of such an individualized and patient-centered assessment will increase even further.

## Pathogenesis of eosinophilic esophagitis: “epidemiology and insights from environmental factors”

### The epidemiology of eosinophilic esophagitis—an accelerated emergence in the face of a rapidly changing environment

Eosinophilic esophagitis (EoE) refers to a chronic, inflammatory, TH-2-type immune-mediated disease exclusively affecting the esophagus with rapid emergence [1–3] subsequent to its first description almost three decades ago [4, 5]. Until recently, EoE was a representative of orphan diseases. However, both epidemiological studies and everyday clinical practice not only indicate that the disease is relatively common (at least in the western world) [1–3]. EoE has also been recognized as THE leading causes of esophageal dysphagia and food impaction in adults [6], as well as an important underlying etiology of failure to thrive and refusal of adequate food intake in children [7].

Currently, the prevalence of EoE in the western world is around 1 in 2500 individuals (1–9/100,000) [1, 3]. As it is the case with any dramatic increase in incidence of a given rather novel disease, the following question arises: Is there just an increase in physicians’ awareness and therefore a lower threshold to detect the condition that might have always been (and still is) present in a relatively stable epidemiological distribution?

The clear answer is no. Although there has been a moderate increase in the overall rate of esophageal biopsies obtained during upper endoscopy in recent years, there is an incomparably larger increase of the fraction of biopsies from the proximal and distal part of the esophagus with high eosinophilic counts (above the diagnostic threshold of > 15 eosinophils per high-power field) [8, 9]. These dynamics can only be explained by an genuine increase in EoE—in excellent congruency with numerous epidemiological investigations [1, 2, 10, 11].

The association of EoE with atopic disease has been repetitively confirmed, with at least 60% of EoE patients currently or previously suffering from concomitant atopic diseases [2, 12–14]. The crucial importance of environmental factors to impact on the integrity of epithelial barrier function has been recognized and a matter of extensive research in other immune-mediated diseases with predominant TH-2 response, including asthma, allergic rhinitis, atopic dermatitis, or food allergies [15]. Environmental factor most likely are important in developed and developing countries alike [16]; indeed, the pace of changes in environmental factors is likely to be even higher in the latter. Perhaps the most extremely rapid and fundamental change in environmental exposure occurs in immigrants, where an assimilation of the hosting country’s risk for several immune-mediated diseases, e.g., IBD [17] has been observed in immigrants and their offspring, typically within very few decades.

A multitude of factors are assumed to play a role in EoE’s etiopathogenesis, including genetics, altered immune response, and alterations in the dietary composition in specific and environmental factors in general. While the reasons for the steep increase in epidemiology still remain rather elusive, the accelerated emergence of EoE within a relatively short interval of less than three decades in itself is a strong advocate for a crucial role of environmental factors [18] in chronic immune-mediated diseases (including IBD [17, 19], psoriasis [20], or asthma [21]) in general, so as specifically in EoE [22].

### Environment in EoE: established and presumptive factors

It is nowadays widely accepted that EoE in the vast majority of patients represents a distinctive form of food allergy [23, 24]. Therefore, it may be hypothesized that a considerable fraction of all environmental factors a given individual is exposed to in conjunction with the global geographic distribution of these factors might represent a proxy of variance in food intake (i.e., type and composition of dietary components). Currently, there is an important limitation regarding the available literature on environmental factors’ potential role in EoE. Most of the research was conducted in relatively circumscribed geographic regions, such as the USA (entire country or only cohorts from circumscribed regions, e.g., North Carolina) or Spain, representing only a small fraction of those regions of the world, where EoE is common and evidently are not at all representative for regions with (at least for the time being) low prevalence. To put this limitation in a nutshell: While as an example brick exterior housing [25] may provide insights for the situation in North Carolina and other western countries alike, we are evidently in need for future research on potential causative environmental factors in regions of the world, where brick exterior in housing are entirely inexistent.

While the aggregation of cases of EoE appears to be high within families, studies in twins and families rather support the predominant role of concomitant environmental exposure as compared to genetic heritability [18].

Environmental factors may even play a role as early as prior to birth. Maternal fever during pregnancy (adjusted odds ratio, aOR 3.18) as well as preterm labor and c-section (aOR 2.18 and 1.77, respectively) were found to be associated with developing EoE in the future [26, 27]. Furthermore, antibiotics in infancy might increase the risk of EoE in adulthood (OR 4.46) [27].

Eosinophilic esophagitis may be more common in rural regions with a lower population density [28], which appears somewhat counter-intuitive, as IBD [29] and atopic diseases appear to be more frequent in urban living conditions with presumed protective effects of frequent childhood contact with farm animals [30]. Rather in line with the hygiene hypothesis [31] on the other hand and in contrast to the former observation—gastric *Helicobacter pylori* infection has consistently been shown to be negatively associated with EoE in a multitude of different investigations [32–34]. In addition, having had a (furry) pet in infancy was found with a considerably reduced associated risk of EoE (aOR 0.58) [26]. Interestingly, EoE may be considered as a rather late landmark in the atopic march’s stretch of way [35].

Aside from typical hygiene aspects, housing conditions might be of additional importance, as EoE was independently associated with living in a house with brick exterior (aOR 1.83) [25].

There are discrepant results regarding seasonal differences in EoE. While a large US case–control study identified a small but consistent seasonal increase in EoE incidence in summer (pointing to the potential role of pollen/aeroallergens) [36], a recent large meta-analysis from Spain did neither observe variations in incidence nor clinical recurrence [37]. A study from the US indicated, that individuals living in a cold climate zone had a roughly twofold increased risk of dysphagia, esophageal eosinophilia. and eosinophilic microabscesses [38].

EoE is increasingly recognized in developing countries or—when it comes to chronic immune-mediated disease typically associated with westernized living conditions—rather untypical regions of the world, including for instance Saudi Arabia [16].

While smoking is the best-studied environmental factor in inflammatory bowel disease, surprisingly little is known on the potential role of smoking on the occurrence of course of disease in EoE. In a case–control study, smoking independently decreased the risk of suffering from EoE in multivariate analysis (OR 0.47) [39]. Contrary, to what one might expect according to the robust evidence in Crohn’s disease, no increased risk of fibrostenotic disease was observed among smoking EoE patients [39]. Furthermore, no significant impact of alcohol intake (even with daily reported intake) on the risk of EoE was observed [39].

Current intake of NSAIDs was found to be associated with a decreased risk in EoE [39], whereas prior intake of antibiotics and PPI use in infancy revealed a risk increase of EoE in childhood (aOR 2.3 and 6.05, respectively) [26, 27, 40].

### Looking at environment in EoE and learn about its pathogenesis?

Although the importance of environmental factors in the pathogenesis of EoE has increasingly received recognition in recent years, discrepantly little knowledge and research is currently available in comparison to for instance genetics [22, 41]. This indicates, that a re-evaluation of the scientific community’s research priorities might be worthwhile and a more intensive exploration of environmental factors might harbor the potential to advance our understanding of EoE in general and its recent dramatic epidemiological rise in specific. Evidently, the underlying reason—it might be indeed more appropriate to assume that there are several potentially synergistic reasons, with distinctive and variable importance according to individual patients—for the steep rise in EoE’s incidence still remain speculative [42]. However, the rapidly changing environment and complexity of EoE patients’ exposures to environmental influences throughout lifetime render it likely that an increased investigations of the environment might be fruitful to advance our understanding of the pathogenesis of EoE.

To conclude, in a disease as multifactorial as EoE, it certainly is plausible to increase our attention, dedication, and scientific focus on a key component of its pathogenesis [43], which is the environment.

## Typical clinical presentation, diagnosis, and treatment of eosinophilic esophagitis

### Definition and characterization of eosinophilic esophagitis

Since the first description of eosinophilic esophagitis (EoE) in the early nineties of the twentieth century, this syndrome is defined as a *clinico-pathological* entity [4, 5]. Because the esophagus is a hidden organ and because this particular inflammatory condition seldom provokes relevant systemic effects, the *clinical part* of its dichotomous definition consists almost exclusively of symptoms. Clinical findings are mainly unremarkable and as such physical examination and laboratory analyses do not in general exhibit significant abnormalities [44]. The *pathological part* of EoE’s definition relies on the histological examination of esophageal biopsy specimens, showing a predominant infiltration with eosinophils [44]. Of note, as a transient eosinophilic tissue infiltration may occur under many circumstances, chronicity is a further element required for diagnosing EoE [45].

### Clinical manifestation of EoE in adults and children

According to the guidelines, “symptoms of esophageal dysfunction” is the umbrella-term of EoE’s clinical manifestation [46]. In adults and adolescents, dysphagia for solids up to food impaction is the leading symptom occurring in almost 95% of patients [44]. However, non-swallowing-related chest pain—occurring either spontaneously or induced by particular maneuvers such as physical exercise or drinking of alcoholic and/or sour beverages—is reported in more than half of adult and adolescent patients [47]. In contrast, children present a much broader spectrum of symptoms, whereas food refusal, chest pain, abdominal pain, diarrhea, regurgitation, vomiting, and failure to thrive are the leading ones [47]. This non-specific presentation requires a proper differential diagnosis including gastroesophageal reflux, celiac disease, intestinal malformations, Crohn’s disease, food allergies, and functional disorders. For pediatricians, diagnosing EoE is therefore a much bigger challenge as for adult gastroenterologists.

### Endoscopic manifestations of EoE

**Fig. 1:**
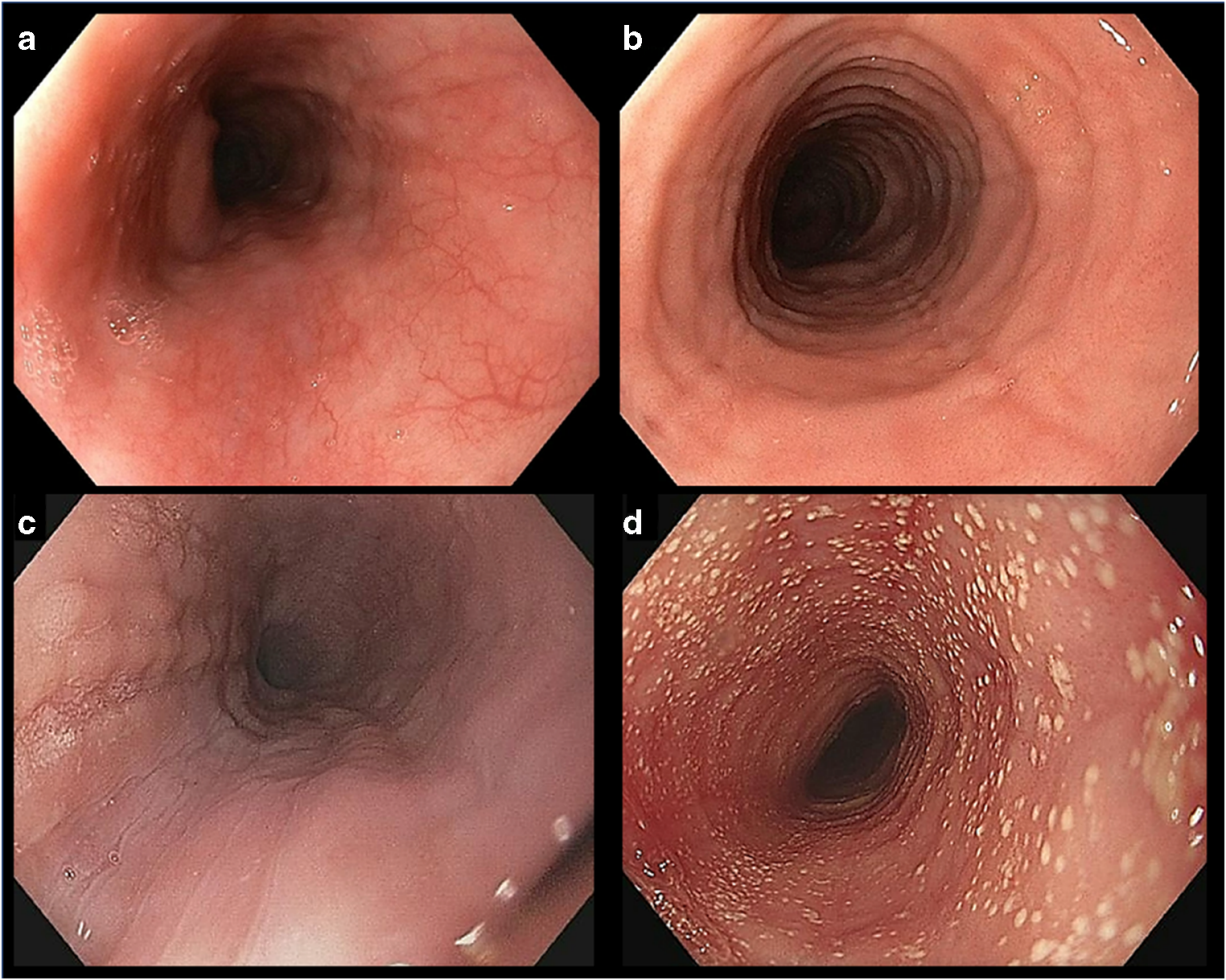
Typical endoscopic features of EoE. Edema may often only be a subtle and patchy finding as illustrated in **a**, where submucosal vessels are readily visible in the right (no edema) but not in the left part (edema present) of the picture. More pronounced edema, rings, and furrows are depicted in **b**, whereas in **c** furrows are more severe with few exudates, which frequently tend to be located within or adjacent to furrows. Extensive white exudates covering nearly the entire surface of the esophagus are shown in **d**

Although there is no pathognomonic endoscopic sign associated with EoE, upper endoscopy is the first step in the evaluation of a patient with solid food dysphagia [48]. A considerable number of different endoscopic features are associated with EoE, the leading ones being longitudinal furrowing, white exudates, edema, long- and short-segment stricture Schatzki ring, corrugated rings, and crêpe paper mucosa [49] (Fig. 1). These signs usually appear in random combination in any given patient. White exudates, edema, and longitudinal furrowing reflect acute inflammation, whereas rings and strictures are a consequence of fibrosis due to a long-standing eosinophilic inflammation. In order to standardize the assessment of the endoscopic manifestations, endoscopists use the so-called EREFS classification, which includes the five major signs [50]. Important, all these signs are suggestive of the diagnosis of EoE, but the endoscopic suspicion requires histologic confirmation. There are even some reports of normal appearing mucosa in EoE. A history of solid food dysphagia requires therefore always a structured biopsy sampling even in the absence of obvious endoscopic abnormalities.

### Histo-pathological manifestation of EoE

A relevant infiltration of the esophageal epithelium with eosinophils is the histological hallmark of EoE [44]. The eosinophils are typically accumulated beneath the surface of the epithelium, often agglomerated to so-called microabscesses and sometimes already degranulated [51]. However, EoE is not a single-cell disease and numbers of different sub-sets of lymphocytes and mast cells are increased as well in the inflamed mucosa of the esophagus. The further characterization of the inflammatory cells and of the mediators has shown that EoE exerts mainly a Th2 inflammatory pattern [52]. In addition to the cellular infiltration, several non-cellular, structural abnormalities occur in EoE. Dilated intercellular spaces, surface layering, basal zone hyperplasia, and papillary elongation are alterations associated with acute inflammation and with an increased cellular turnover. The eosinophils are late-phase inflammatory cells with powerful repair properties [53]. A chronic inflammation leads therefore to fibrosis of the involved organ [54]. In EoE—in contrast to reflux disease—in the lamina propria usually a substantial fibrosis can be seen [55]. The eight leading EoE-associated alterations are summarized and graded in a histological scoring system, called EoE-HSS [51]. It is recommended using this instrument when evaluating esophageal biopsies for the purpose of randomized clinical trials. However, in observational studies or regular clinical practice, performing EoE-HSS on a routine basis may be too cumbersome.

### Diagnostic criteria of EoE

Based on the above-mentioned considerations, a firm diagnosis of eosinophilic esophagitis requires the following three items:

- A prolonged existence of symptoms attributed to esophageal dysfunction
- An eosinophil predominant infiltration of the esophageal epithelium with peak values of more or equal than 15 eosinophils per high-power field (HPF)
- An exclusion of other reasons for esophageal tissue eosinophilia, mainly reflux disease, eosinophilic gastroenteritis, Crohn’s disease, and connective tissue disorders [44]

Of note, since the recognition that PPI-responsive eosinophilia is nothing else than typical EoE with the only exception that it responds in addition to the established treatments to PPI as well [56], the diagnostic PPI-trial is obsolete and therefore not anymore requested for establishing the diagnosis [57].

### Natural history and complications of EoE

When confronted with a new disease, the understanding of the natural history is crucial for making any decisions regarding monitoring and treatment. Despite the fact that our understanding of the natural history of EoE is still limited, it has become clear that it is a chronic disease with either chronic-persistent or chronic-relapsing course [45]. Currently, EoE seems to not harbor an increased risk for malignancies, but long-term follow-up is required to confirm this observation. The main concern is that a long-standing, untreated eosinophilic inflammation leads to fibrosis with wall thickening, abnormal fragility and strictures, finally evoking a structural and functional damage of the esophagus [55, 58]. This so-called remodeling [59] predisposes to several complications: Acute food bolus impaction is the leading complication of untreated EoE. During the course of their disease, more than one-third of adult patients will experience long-lasting food impactions with the necessity for endoscopic removal [60]. This frightening and risky complication is a sword of Damocles hanging permanently over almost all EoE patients. Long-lasting impactions harbor the risk for retching-induced esophageal rupture (Boerhaave’s syndrome), procedure-related perforation and aspiration pneumonia [60]. In addition, remodeling can impair the function of the lower esophageal sphincter (LES) leading to a secondary reflux. In a case series, 10 of 26 (38%) adult EoE patients had pH monitoring confirmed coexisting reflux disease [49]. Finally, we have to admit that our knowledge of the natural history of EoE is still fragmentary.

### Goals of and indications for treatment

As a consequence of EoE’s dichotomous definition, the primary therapeutic goal is a dichotomous as well: resolution of symptoms *and* control of the inflammation [47]. From the patients perspective resolution of symptoms is the main treatment target[61]. However, as an ongoing eosinophilic inflammation results in a long-term organ damage, control of eosinophilic inflammation has to be regarded as an at least as important therapeutic goal. A cut-off of less than 15 eosinophils per high-power field has shown to be an appropriate endpoint in several therapeutic trials [57]. In conclusion, the ideal therapeutic intervention in EoE has to improve symptoms as well as to control the inflammation.

### Therapeutic options: drugs, diet, and dilation (DDD)

EoE is in the vast majority of patients an allergic response induced by food proteins, but shares in addition similarities with gastroesophageal reflux disease. Drugs used to treat allergies and reflux disease have therefore intensively been tested as medical treatments for EoE. However, if the causative food can be identified an elimination diet is an attractive, non-medical treatment option. Finally, in refractory EoE, dilation can be needed. The therapeutic options can therefore be summarized as the 3 D’s: drugs, diet, and dilation [47].

### Medical treatment of eosinophilic esophagitis

#### Corticosteroids

In the first study demonstrating that systemic corticosteroids are able to bring active EoE into remission, a series of pediatric patients with esophageal eosinophilia and chronic reflux-like symptoms unresponsive to aggressive anti-reflux therapy were treated with methylprednisolone [62]. After corticosteroid therapy, all but one patient had dramatic clinical and histological improvement. Shortly afterwards, it was demonstrated that EoE patients responded even to treatment with swallowed, topically acting corticosteroids (STC) [63]. Ten years later, a prospective controlled trial comparing prednisone with swallowed fluticasone showed that STCs were as effective as systemic corticosteroids but had fewer side effects [64]. This study provided the first strong evidence that EoE can be controlled without the use of systemic corticosteroids. Meanwhile, a substantial number of controlled clinical trials have confirmed that STCs such as budesonide, fluticasone, ciclesonide, or mometasone deposited on the esophageal surface are highly efficient in adult and pediatric EoE patients in bringing active EoE clinically and histologically in remission [47]. Overall, the response rates with STCs were between 50 and 93%. The main drawback of corticosteroid treatment is that almost all patients rapidly relapse after cessation of therapy [65, 66]. Therefore, corticosteroids are able to control, but not to cure EoE. The main side effect of STCs is colonization with *Candida albicans*, occurring in 10 to 15% of the patients. This infection is often asymptomatic and requires only a therapy in case of symptoms. Respecting the well-documented efficacy and the favorable safety profile, the European Medicines Agency EMA has approved the first budesonide preparation—an oral-dispersible budesonide tablet specifically designed for the esophageal administration (Procedure No. EMEA/H/C/004655/0000)—for the treatment of EoE. Since EoE is a chronic disease and relapses occur rapidly after cessation of an induction treatment, a maintenance therapy is required. Subsequently, two controlled long-term studies using budesonide have demonstrated that this compound is able to maintain a clinico-histological remission in up to 75% of the patients, whereas less than 5% of the participants under placebo were still in remission after one year of treatment [67]. Of note, as four of the total 136 patients (≈3 % ) exposed to budesonide had decreased morning cortisol levels, further studies evaluating the safety of the long-term use of STCs are needed. Today, it is still prudent to exercise caution, when treating children and adults with high doses of swallowed topical corticosteroids for longer time periods.

#### Proton pump inhibitors

Proton pump inhibitors (PPI) are recommended in EoE patients with coexisting gastroesophageal reflux disease (GERD) [44]. Given the high prevalence of GERD in a general population, it is likely that both diseases can coexist. However, the interplay between EoE and GERD is more complex [68] and it is also possible that an impairment of the lower esophageal sphincter may lead to secondary reflux and that a hypersensitivity of the eosinophil-inflamed esophageal mucosa could provoke reflux-like symptoms even in absence of obvious reflux. Moreover, it is meanwhile established that a subgroup of EoE patients, initially called PPI-responsive esophageal eosinophilia, responds to PPI even in the absence of reflux. Although real-world data suggest STC the most effective treatment option, PPI are much more frequently prescribed as first line therapy than STC (76.4% vs. 10.5%) according to a European registry, predominantly with patients from Spain [69]. Furthermore, it remains to be determined where PPIs must be positioned in the treatment algorithm of EoE, because so far no robust studies directly comparing the efficacy and long-term safety of PPI with STC drugs are available.

#### Immunosuppressants (thiopurines)

As already mentioned, in on average 70% of patients, remission can be achieved with STCs. In other words, roughly 30% of EoE patients do not achieve the therapeutic goals despite properly performed treatment with STCs. In analogy to steroid-refractory inflammatory bowel disease, immunosuppressants have been evaluated in these patients. Indeed, in a small series of adult patients with refractory EoE. purine analogues have shown promising results [70]. Surprisingly, this treatment modality has not been further evaluated.

#### Biologics

Interleukin 5 (IL-5) plays a critical role in maturation, differentiation, activation, and tissue recruitment of eosinophils. Mepolizumab and reslizumab—both humanized anti-IL-5 antibodies—are highly specific anti-eosinophil drugs. Three controlled trials with these anti-IL-5 blockers have demonstrated a significant reduction of eosinophils in the esophageal tissue and in the peripheral blood [71–73]. Unfortunately, both substances exerted only minimal clinical benefits in all three studies. However, it currently remains unclear, whether use of non-validated outcome instruments in these trials are at least in part associated with the observed lack of clinical improvement. Because IL-4 and IL-13 play a key role in the pathogenesis of EoE as well, treatment with monoclonal antibodies blocking these two cytokines were evaluated too. RPC4046, a pure IL-13 blocking compound, and dupilumab, a combined blocker of the IL-13 receptor and the alpha-unit of the IL-4 receptor, have shown in phase 2 trials promising results [74, 75]. Both drugs are currently further evaluated in phase 3 trials. In conclusion, among the already evaluated biologics, IL-13 and IL-4 blockers appear to represent the most promising approach in patients with refractory EoE.

### Dietary treatment of eosinophilic esophagitis

Eosinophilic esophagitis is mainly induced by food proteins. Avoidance of a contact between the esophageal surface and food proteins has demonstrated to reliably resolve symptoms and inflammation, opening a door toward a causative non-medical treatment [76]. Currently, the challenge is to identify both the culprit food categories and those patients who are suited for a dietary intervention. As IgE’s do not play a key role in inducing the inflammatory response [77], IgE-based tests such as skin prick tests or determination of food-specific serum IgE’s are not reliable in the search for causative nutrition. In general, the following principal dietary approaches can be differentiated:

#### Elemental diet

Twenty-five years ago, a pediatric case series has shown that a protein-free diet using an acid-based formula is able to bring more than 90% of EoE patients in remission [76]. This so-called elemental diet is therefore currently the most efficient dietary regimen. Nevertheless, taste and costs of the formulations are markedly affecting the quality of life of the patients. Currently, there are two potential indications, namely, in patients refractory to medical and less restrictive dietary treatments as well as a radical initial modality to enforce a remission in severely diseased patients.

#### Targeted elimination diet

The identification of the culprit food group(s) and consecutive individualized elimination would represent the most stringent and elegant treatment for EoE. Unfortunately, the validity of different methods of allergy testing—for instance, skin prick tests, atopy patch tests, serum IgE, microarrays—to identify culprit food categories is disappointingly low because the majority of these tests is IgE-based and IgE’s play in EoE only a subsidiary role [77]. The targeted elimination diet is therefore, as long as no meaningful diagnostic tools are available, of limited value.

#### Empiric elimination diet

Empiric elimination diet turns the perspective from the patient to the risk of particular food categorizes provoking allergic responses. Starting with the elimination of the six most critical food categories (6-FED)—milk, gluten, egg, soy, peanut/tree nuts, fish, and seafood—followed by stepwise and controlled re-introduction (step-down strategy) is currently the best evaluated dietary treatment modality in EoE. According to a meta-analysis, remission in about three quarters of patients can be expected [78]. Because the controlled re-introduction requires serial endoscopies, this form of dietary treatment is cost- and time-intensive. The most frequently identified culprit foods are milk, gluten, and eggs. In addition, frequently only one or two of the six foods were identified as triggers [79, 80]. This suggests that less comprehensive forms of dietary restriction might be comparably effective. Indeed, a shift of paradigm starting with a 1- or 2-FED followed by a step-up strategy is increasingly advocated and has shown first promising results [81]. This approach is easier to follow and might increase the acceptance of dietary treatment approaches in both patients and physicians.

### Treatment of eosinophilic esophagitis with dilation

A chronic, untreated eosinophilic inflammation induces fibrosis leading to wall thickening, loss of elasticity and stricture formation [54, 55]. Beside the acute inflammation, this so-called remodeling can create symptoms and in addition is a risk for food bolus impactions. Because the response of this fibrosis to anti-inflammatory treatment is slow, however, dilation is the treatment of choice when symptoms persist despite successful treatment of the inflammation. Important, the underlying inflammation is not influenced by this procedure [82]. Summarized, dilation is an efficient measure to control symptoms but should be used as second-line treatment and only in combination with a medical or dietary anti-inflammatory treatment.

## Atypical presentation of eosinophilic esophagitis

### EoE beyond esophageal eosinophilia

Esophageal eosinophilia is the hallmark of EoE; an eosinophilic infiltration of at least 15 eosinophils per high-power field is required to correctly and unambiguously establish the diagnosis [14]. However, the pivotal role of eosinophils has been questioned lately. The only modest correlation between symptoms and esophageal eosinophilia suggests the presence of other pathogenic mechanisms beyond pure eosinophilic infiltration [83]. Indeed, the newly developed EoE histology scoring system that takes into account various histologic changes (such as basal zone hyperplasia, spongiosis, and lamina propria fibrosis) appears to outperform peak eosinophil counts for both EoE diagnosis and monitoring [51]. In addition, eosinophil-targeted treatment with the monoclonal antibodies mepolizumab and reslizumab—despite achieving dramatic reduction in eosinophil counts—did not induce clinical response [71–73]. Therefore, adhering to an arbitrary chosen cut-off value (15 eos/hpf) to dichotomize patients into having EoE vs. having no disease is probably too simple. As it is known for other diseases, subtypes or variants might exist unraveling EoE as a spectrum disorder. The classical EoE with a significant eosinophilic infiltration might only be the tip of the iceberg representing the most extreme and most appealing phenotype of this disease spectrum.

### EoE-like disease

In Switzerland, a new EoE-like phenomenon termed as EoE-like disease has been described in 2016 [84]. Five patients (four females, one male) from four families with known EoE were evaluated for typical EoE symptoms (dysphagia for solids, food impactions, and chest pain), but without esophageal eosinophilia upon histological examination. These symptoms persisted despite high-dose PPI treatment, but responded rapidly to swallowed topical steroids (clinical remission in 4/5). While endoscopic changes were only subtle (slight mucosal irregularities such as fine nodules and rings), these patients showed T cell infiltration (CRTH2 negative, not fulfilling criteria for lymphocytic esophagitis) and moderate mast cell infiltration, and gene expression abnormalities similar to classical EoE (such as MUC4 and CDH26 genes), suggesting a uniform underlying pathogenesis [84]. Motility abnormalities and GERD, two potential differential diagnoses, were excluded by high-resolution manometry and 24-h impedance pH monitoring in 3/5 patients. Of note, expert pathology review of histologic slides (hematoxylin and eosin staining) showed minor lymphocytic infiltration and signs of chronic inflammation captured by the EoE histology scoring system such as basal zone hyperplasia and papillary elongation. The latter suggests that even with simple histology patients with EoE-like disease might be identified and distinguished from patients without esophageal pathologies.

**Fig. 2:**
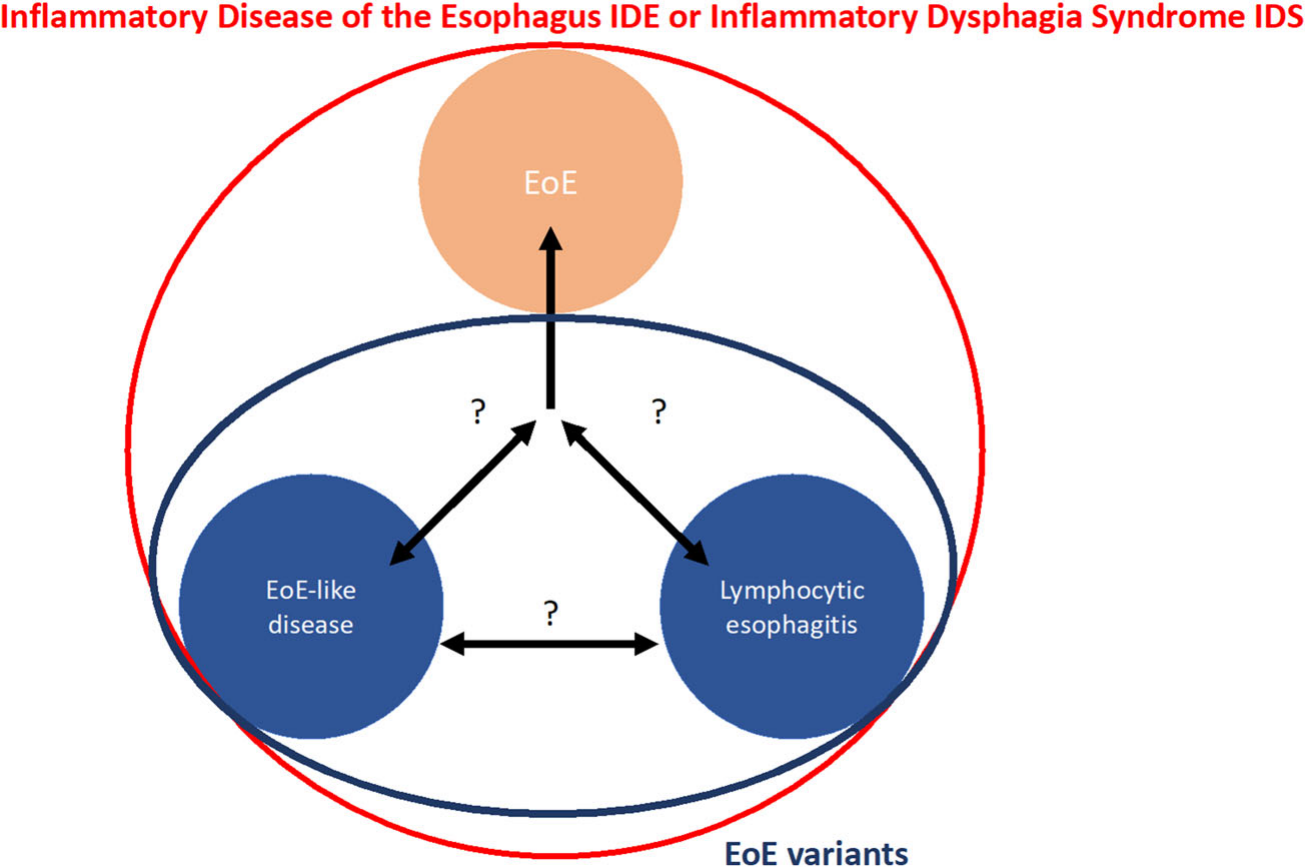
Potential classification of EoE-like disease and lymphocytic esophagitis as EoE variants within the EoE spectrum. The term inflammatory disease of the esophagus IDE or inflammatory dysphagia syndrome IDS might be used to describe this disease spectrum

A multicenter observational study revealed indeed a high proportion of EoE-like disease patients with presence of EoE histology scoring system features, CRTH2-negative T cell infiltration and disturbed epithelial integrity [85]. The latter might indicate—as it is known for EoE—an epithelial barrier defect enabling allergen translocation and promotion of inflammation. Intriguingly, in 8 patients, progression from EoE-like disease to classical EoE was observed after a median of 14.0 months (IQR 3.6–37.6) supporting the hypothesis of a broader spectrum disease (such as inflammatory dysphagia syndrome IDS or inflammatory disease of the esophagus IDE), where EoE presents the most extreme variant (Fig. 2).

### Lymphocytic esophagitis

The term lymphocytic esophagitis has first been coined by Rubio et al. in 2006 [86]. The authors reported on 20 patients with chronic esophagitis showing “high numbers of intraepithelial lymphocytes gathered mainly around peripapillary fields and by none to occasional intraepithelial granulocyte.” Fifteen years later, the entity of lymphocytic esophagitis remains poorly defined [87]. In contrast to EoE, patients tend to be older and more often females; particularly females in their sixth decade appear to be affected [88–90]. However, the clinical presentation with solid food dysphagia being the most common symptom is indistinguishable from that of EoE. Other lymphocytic esophagitis symptoms such as chest pain, heartburn, nausea, and abdominal pain are also known from patients with EoE [88–91]. As it is seen for EoE-like disease, endoscopic findings are commonly subtle or absent (in up to one-third endoscopy has been reported as completely normal). Still, EoE typical inflammatory and/or fibrotic signs (furrows, rings) can be observed [88, 90, 91]. Histologically, the diagnosis is based on the original description by Rubio and colleagues [86], defined as typical pattern with high numbers of intraepithelial lymphocytes (usually more than 20–30 per hpf), gathered mainly in peripapillary fields, peripapillary spongiosis (dilated intercellular spaces), and absence of intraepithelial granulocytes [92].

As large long-term follow-up studies and molecular characterization are missing, it remains unknown if there is an overlap between lymphocytic esophagitis and EoE or if they represent two completely distinct and mutually exclusive disorders. At least, preliminary results from the aforementioned multicenter study on EoE-like disease suggest some possible overlaps with regards to mRNA profiles and also some transition/progression between lymphocytic esophagitis, EoE-like disease, and EoE [85].

### Clinical implications of EoE variants and future perspectives

EoE-like disease and lymphocytic esophagitis share the same clinical presentation as EoE, while the EoE typical esophageal eosinophilia is absent. Still, histologic changes can be detected based on the EoE histology scoring system and intraepithelial lymphocytes. Molecular analyses and first follow-up studies suggest some potential pathogenic overlap (Fig. 1). Long-term follow-up studies with inclusion of patients at various expert centers, together with in-depth big data analyses will pave the role toward a more detailed characterization of these entities. Of particular interest will be the question whether EoE-like disease and lymphocytic esophagitis are—like EoE—food allergen mediated. Epithelial barrier dysfunction at least implicates a potential role of such allergens entering the esophageal mucosa.

Knowledge of EoE variants is crucial as otherwise patients not fulfilling the diagnostic cut-off criteria for EoE may end up as being considered as having functional disease. Histological evaluations (plus immunohistochemistry and mRNA profiles) however clearly show that this is not the case. Whether these conditions represent distinct entities, subtypes, or variants of EoE, or early stage or burned out EoE remains to be determined. Investigation of these conditions will not only likely answer such uncertainties but also help to gain pathogenic insights into an EoE pathophysiology beyond eosinophilic infiltration and thereby pave the road to novel non-eosinophil-targeted medications.

## Differential diagnosis of esophageal eosinophilia

Although EoE represents the epitome of eosinophilic infiltration in the esophageal tissue, esophageal eosinophilia (EE) has been described in many other diseases. In fact, to diagnose EoE accurately, in addition to symptoms related to esophageal dysfunction and an eosinophilic count ≥ 15 eos/hpf, differential diagnosis must be excluded [14, 57, 93, 94]. However, since these differential diagnoses are themselves often associated with EoE, another reason of EE never excludes a concomitant EoE.

Unlike in other part of the gastrointestinal (GI) tract [95, 96], eosinophils are not normal inhabitants of the esophageal mucosa. Hence, an EE should result in a diagnostic workup. In this review, we summarize the latest data about EE other than EoE.

### Gastroesophageal reflux disease

One of the most common causes of EE is GERD. When faced with the question of whether a GERD or an EoE is present, several things must be considered. First of all, it should be emphasized that GERD and EoE can coexist and are not mutually exclusive. In a patient with an erosive esophagitis and a pathological 24-h pH-metry, a diagnosis of GERD may be established [97], but this never excludes a coexisting EoE. Secondly, there exist no criterion to distinguish GERD from EoE. Clinically, they share similar symptoms [98], endoscopic features are not specific for EoE [99], and a high number of eosinophilic count (eos/hpf) may be also present in GERD [100]. However, an eosinophil count (eos/hpf) lower than 5 is highly suggestive of GERD [101], but does not exclude EoE reliably [102]. Thirdly, patients responding to PPI therapy are indistinguishable from EoE [14, 57, 103]. Hence, PPIs are currently considered as treatment option in EoE and not as a diagnostic criterion [104, 105]. Having discussed all the hurdles above, how can we make a distinction between GERD and EoE in daily life? Since there exists no test to rule out GERD, the clinical presentation of the patient is key and a precise history of symptoms is of crucial importance. An interesting approach is suggested by Cotton et al., who proposed a diagnostic model for diagnosing EoE demonstrating dysphagia as the most reliable predictor [106].

### Celiac disease (CeD)

Former studies suggested an association between celiac disease and EoE/EE with an up to 10% prevalence of EE in CeD patients and, inversely, a prevalence of CeD in EoE patients [107–112]. However, newer data are more conflicting [113] and refute these former findings [112, 114, 115]. The higher incidence of EE in CeD patients may be explained due to referral and selection bias. A recently published study demonstrated a prevalence of around 1% of EE in CeD patients [116]. Furthermore, HLA DQ2 and DQ8 haplotypes are not increased in adult EoE patients compared to the general population [117]. Hence, there is hardly any evidence supporting EE being a CeD-associated finding [118] and not more common than in the general population. The recommendation to obtain duodenal biopsies to rule out CeD in patients with EE is therefore questionable.

### Inflammatory bowel disease

Most guidelines suggest excluding IBD as a cause of EE [14, 94]. However, EE in Crohn’s disease and ulcerative colitis is rare [119–122] and is accompanied nearly always with an inflammation in stomach and duodenum. Moreover, the inflammation in the esophagus is unspecific [123, 124] and rather has a lymphocytic than an eosinophilic pattern [125, 126]. Although a recent publication showed a fivefold increased risk of EE in IBD [127], it is possible that most of these patients had rather an EoE than an isolated EE. Large epidemiologic data support a positive association between IBD and EoE with a up to fivefold increased risk of EoE in IBD patients and inversely an up to sixfold increased risk of IBD among EoE patients [128]. Interestingly, but not in line with the former data [128], a study investigating a large database could not show a relationship between UC and EoE, but even found a significant inverse relationship between EoE and CD [129]. Nevertheless, since isolated EE in IBD is rare, a precise medical history about swallowing difficulties is key to not miss a concomitant EoE.

### Motility disorders

Many case series indicate that achalasia can lead to EE, mostly with only small numbers of eosinophils in the mucosa, but higher eosinophilia in the muscularis propria [100, 130, 131]. Inversely, it is well-known that EoE may lead to motility dysfunction [132–134] making it difficult to distinguish whether the eosinophilic infiltration is caused by achalasia or vice versa.

### Autoimmune connective tissue disease

Although most connective tissue diseases, such as eosinophilic granulomatosis with polyangiitis, lupus erythematodes, rheumatoid arthritis, systemic sclerosis, Sjögren syndrome, or scleromyositis may cause tissue eosinophilia in the GI tract, it occurs mostly in the small bowel, stomach, or colon [135, 136]. An esophageal involvement is a rarity [136, 137] and as opposed to EoE, most patients are female, have no atopy, and present with peripheral eosinophilia [136].

### Autoimmune skin diseases

In case of an oral or esophageal involvement, pemphigus vulgaris (PV) [138, 139] and bullous pemphigoid (BP) [140, 141] may present with similar symptoms to EoE, namely, dysphagia and EE. However, the typical histology with linear deposits of IgG and/or C3 along the basement membrane in BP and in the epidermis in PV as well as the involvement of the skin, facilitates a distinction between an EoE and an esophageal involvement of PV or BP.

### Infections

A hallmark of eosinophils is their role in helminthic infection resulting in a migration to areas of parasitic infection [96]. Although the majority of cases results in an eosinophilic infiltration of the ileum or appendix, case reports found EE in patients with pinworm infection (*Enterobius vermicularis*) [142] and with anisakiasis [143].

### Non-esophageal eosinophilic gastrointestinal disorders, hypereosinophilic syndrome

In case of an involvement of eosinophilic infiltration in other parts of the gastrointestinal tract and absence of known causes of eosinophilia, the disease is classified as eosinophilic gastritis, gastroenteritis, or colitis, irrespective of EE [144]. These non-esophageal eosinophilic disorders (nEGIDs) present with a variety of symptoms based on the affected site (organ) and extent (layer) with or without esophageal involvement [145]. It is noteworthy that an eosinophilic infiltration in the GI tract distal to the esophagus is found physiologically and therefore no defined threshold for the number of eos/hpf in nEGIDs exists [146]. Furthermore, mucosal biopsies may be normal in a muscular or subserosal involvement.

Hypereosinophilic syndrome (HES) is defined as a an eosinophil count more than 1500 cells/ml without secondary causes for at least 1 month and evidence of end organ manifestation attributable to the eosinophilia [147]. It is characterized by multiorgan system infiltration by eosinophils, but may involve esophageal tissue resulting in EE with dysphagia [148, 149].

### Rare causes

Other rare causes of EE are summarized in Tables 1 and 2.

**Table 1:** Overview on reported environmental risk factors for EoE including the magnitude of increase or decrease in risk, whenever available. Associations are marked in the second column as either positive (+), or negative (−) association and with ± in case of mixed or unclear evidence

Environmental factor	Association with EoE: +, −, or ±	Role in EoE
Cold climate zone	+ OR 2.02	OR 2.02: increased risk of dysphagia, esophageal eosinophilia, and eosinophilic microabscesses in cold climate zone [38]
Seasonal variation	±	Unclear. Cross-sectional US investigation with mild increase in diagnosed EoE cases in summer months (July), aOR 1.13 [36]; in contrast, systematic literature review including 18 studies (comprising 16 846 patients) did not find a seasonal variation of neither diagnosis or relapse of EoE [37]
Population density/living in rural areas	+ aOR 1.27	Nationwide US pathology data base. Significant increase in esophageal eosinophilia in subjects with lowest population density (as opposed to highest quintile of population density) [28]
*Helicobacter pylori* infection	− OR 0.24–0.77	German case–control study, seroprevalence OR 0.24 [32]; cross-sectional US study on esophageal eosinophilia OR 0.77 [34]; meta-Analysis of 11 observational studies: 37% and 38% risk reduction in odds of EoE and esophageal eosinophilia, respectively [33]
*Herpes simplex* infection of the esophagus	±	Several case reports and case series in pediatric and adult patients of patients with HSV esophagitis and primary diagnosis of EoE (e.g., [150, 151]). Unclear if causal association or rather a prior diagnosis of EoE as risk factor for HSV esophagitis
Brick exterior housing	+ aOR 1.83	Patients with EoE more frequently living in houses with a brick exterior, forced air, or gas heating. Brick exteriors revealed to be independently associated with EoE [25]
Smoking	− aOR 0.47	Case–control study (USA). Lower likelihood of ever smoking in EoE cases vs. control, no association of smoking with fibrostenosis of the esophagus in EoE patients. No association with alcohol consumption in multivariate analysis [39]
Furry pet in infancy	− aOR 0.58	Dog or cat [26]
Current NSAID intake	− aOR 0.36	Case–control study (USA) [39]
Antibiotics in infancy	+ aOR 2.30	[26]; aOR 3.51 [27]
Acid suppressants in infancy	+ aOR 6.05	Similar association if restricting to symptoms at age ≥ 3 years or older (aOR, 6.05) [26]; use of PPI in infancy positively associated with later diagnosis of EoE: PPI (5.7% EoE cases vs. 1.6% controls; *P* < 0.0001) [40]
Maternal fever during pregnancy	+ aOR 3.18	Maternally reported, potential cluster during 2nd trimester [26]
Preterm labor	+ aOR 2.18	Non-significant association (CI, 0.71–2.72) [26] and aOR 2.56 (non-significant) [27]
C-section	+ aOR 2.18	[26]; aOR 6.21 but non-significant association [27]
Breastfeeding and genetic susceptibility	− aOR 0.08	Breastfeeding in conjunction with rs6736278 (CAPN14) [41]; this investigation indicates that there may be an important interplay of environmental and genetic factors!

**Table 2:** Overview on environmental factors in eosinophilic esophagitis

Diseases with esophageal eosinophilia	Special remarks
Gastrooesophageal reflux disease	Thorough medical history is key; dysphagia as the most reliable predictor for EoE and not GERD
Celiac disease	Recent studies doubt an association between EE and CD
Inflammatory bowel disease	Think of EoE and not isolated EE in CD or UC
Achalasia	Questions remain whether achalasia causes eosinophilic infiltration or vice versa
Autoimmune connective tissue disease	Esophageal involvement is a rarity
Bullous pemphigoid, Pemphigus vulgaris	Typical histology and involvement of skin
Infections (especially helminthes)	Mostly with small bowel involvement
Eosinophilic gastritis, gastroenteritis and colitis	All disorders may present with esophageal involvement but are classified as EG, EGE of EC based on the affected GI site
Hypereosinophilic syndrome	Peripheral blood eosinophils > 1500 cells/ml without secondary causes and evidence of an eosinophil-mediated end organ manifestation
Atopic diseases (Asthma, aspirin-exacerbated respiratory disease, IgE mediated food allergy) [152, 153]	Extraintestinal manifestations
Drug hypersensitivity and pill esophagitis	Medical history
Radiofrequency ablation for Barrett’s esophagus [154–156]	Typically without dysphagia
Graft-versus-host disease [157]	Eosinophil density correlates with GvHR
Oral immunotherapy [158]	Mostly transient and without symptoms
Asymptomatic esophageal eosinophilia [159–162]	Exclusive diagnosis

In summary, the most common disease associated with EE besides EoE is GERD. Therefore, before ruling out very rare diseases, a precise medical history is key and leads mostly to the correct diagnosis.
